# Characterization of the interactions of chemically-modified therapeutic nucleic acids with plasma proteins using a fluorescence polarization assay

**DOI:** 10.1093/nar/gky1260

**Published:** 2018-12-19

**Authors:** Hans J Gaus, Ruchi Gupta, Alfred E Chappell, Michael E Østergaard, Eric E Swayze, Punit P Seth

**Affiliations:** Ionis Pharmaceuticals Inc., 2855 Gazelle Court, Carlsbad, CA 92010, USA

## Abstract

Interactions of chemically modified nucleic acid therapeutics with plasma proteins play an important role in facilitating distribution from the injection site to peripheral tissues by reducing renal clearance. Despite the importance of these interactions, analytical methods that can characterize binding constants with individual plasma proteins in a reliable and high throughput manner are not easily available. We developed a fluorescence polarization (FP) based assay and measured binding constants for the 25 most abundant human plasma proteins with phosphorothioate (PS) modified antisense oligonucleotides (ASOs). We evaluated the influence of sequence, sugar modifications, and PS content on ASO interactions with several abundant human plasma proteins and determined the effect of salt and pH on these interactions. PS ASOs were found to associate predominantly with albumin and histidine-rich glycoprotein (HRG) in mouse and human plasma by size-exclusion chromatography. In contrast, PS ASOs associate predominantly with HRG in monkey plasma because of higher concentrations of this protein in monkeys. Finally, plasma proteins capable of binding PS ASOs in human plasma were confirmed by employing affinity chromatography and proteomics. Our results indicate distinct differences in contributions from the PS backbone, nucleobase composition and oligonucleotide flexibility to protein binding.

## INTRODUCTION

The phosphorothioate (PS) backbone modification, where one of the non-bridging oxygen atoms of the phosphodiester (PO) linkage is replaced with sulfur, represents one of the most widely used chemical modifications in nucleic acid therapeutics (1). PS antisense oligonucleotides (ASOs) show increased resistance to nucleases which stabilizes the ASO from degradation in biological fluids (2). PS ASOs also show enhanced binding to plasma, cell-surface and intracellular proteins which facilitates distribution to peripheral tissues, cellular uptake and intra-cellular trafficking (3). After binding to plasma proteins, PS ASOs circulate transiently in the blood compartment from where they are thought to partition onto to cell-surface proteins (4) and gain entry into cells by endocytic processes (5). While significant progress has been made recently towards understanding cellular uptake of ASOs, including the characterization of several cell surface receptors (6–8), it remains unclear what contribution plasma proteins have for ASO tissue distribution beside limiting glomerular filtration and urinary excretion (9). We recently reported that PS ASOs show 2-fold improved activity in α-2-macroglobulin knockout mice suggesting that interactions with specific plasma proteins can shuttle ASOs into non-productive cellular compartments (10). Furthermore, interactions of PS ASOs with specific plasma proteins can modulate hematological toxicities in the plasma compartment in a species-dependent manner (11). Given this background, a method which allows for characterization of interactions of PS ASOs with individual plasma proteins from the different species used for preclinical development of nucleic acid therapeutics could be beneficial. However, methods to characterize interactions of PS ASOs with plasma proteins have largely been limited to filter binding assays and size exclusion chromatography which do not inform on interactions with individual proteins (12).

To address these issues, we developed a fluorescence polarization (FP) assay to characterize the interactions of PS ASOs with individual plasma proteins. FP-based assays have been applied for a wide range of bioanalytical applications, including clinical and food analysis and environmental monitoring (13). Molecular interactions can be measured by changes in depolarization of the emitted fluorescence upon binding of a labeled analyte with a macromolecule. FP-based assays also allow for measurement of binding constants in solution at equilibrium without the need for surface immobilization of either the proteins or the analyte. In this manuscript, we report an FP assay to measure binding constants of plasma proteins with PS ASOs. The identification of Alexa Fluor 647 as an optimal fluorescent dye and the effect of ASO sequence, chemistry, PS backbone, and strandedness on these interactions is also reported. Lastly, the effect of species on plasma protein binding and the relevance of the interactions with individual proteins was confirmed by ASO-affinity chromatography and proteomics.

## MATERIALS AND METHODS

The ASOs used in the study were designed at Ionis Pharmaceuticals and synthesized at IDT (Coralville, IA, USA) or Ionis Pharmaceuticals utilizing standard protocols. Alexa Fluor 647 from Life Technologies (Gaithersburg, MD, USA) was the fluorescent tag of choice for conjugating the ASOs at the 5′-end. The human albumin was purchased from EMD Millipore (Calbiochem). Albumin from human, rat, mouse, and monkey for the species comparison experiment was purchased from Equitech (Kerville, TX, USA). IgG, Complement factor 3 (C3), transferrin, fibrinogen, alpha-1-acid glycoprotein were purchased from Sigma Aldrich (St. Louis, MO, USA). Apolipoprotein AI, apolipoprotein AII, alpha-1-antitrypsin, haptoglobin, hemopexin, transthyretin, antithrombin III, β-2-glycoprotein, ceruloplasmin, C1q, C4, plasminogen, ApoB100, factor H, Apo E and factor V were purchased from Genway Biotech (San Diego, CA, USA). Alpha-2-macroglobulin and antichymotrypsin were purchased from MyBiosource (San Diego, CA, USA).

Native histidine rich glycoprotein (HRG) was purified from citrated plasma of Balb/c mice, Sprague-Dawley rats, cynomolgus monkey, and humans (BioIVT, Westbury, NY, USA) using a phosphocellulose column as previously described (14). Recombinant mouse and human HRG were acquired from Sino Biologicals (Collegeville, PA, USA). HRG plasma concentrations were determined for each species by western blot using purified and/or recombinant protein standards. HRG plasma concentrations were orthogonally confirmed by ELISA (mouse, human, and rat) and/or LC/MS using isobaric peptide standards (mouse, human, monkey). The peptides utilized for quantitation of HRG from human and monkey plasma have the Peptide Atlas accession numbers PAp00024649 and PAp00043573 (15), and the mouse plasma peptides PAp00399103 and PAp00382579. Isobaric labeled peptides utilized in the LC/MS assay were synthesized at Pierce Biotechnology (Rockford, IL, USA).

Fluorescence polarization experiments were performed using ALEXA647-labeled ASOs synthesized at Integrated DNA Technologies (Coralville, IA, USA). Measurements were performed in 1× phosphate-buffered saline (PBS), except for the experiments to determine salt and pH dependence of binding. For those evaluations a 10 mM phosphate buffer with a sodium chloride concentration of 50–200 mM and a pH of 5, 6 or 7 was utilized. The assay was set up in 96-well Costar plates (black flat-bottomed non-binding) purchased from Corning, NY, USA. Binding was evaluated by adding ALEXA647-labeled ASOs to yield 2 nM concentration to each well containing 100 μl of protein from sub nM to low mM concentration. Readings were taken using the Tecan (Baldwin Park, CA, USA) InfiniteM1000 Pro instrument (λ_ex_ = 635 nm, λ_em_ = 675 nm). Using polarized excitation and emission filters, the instrument measures fluorescence perpendicular to the excitation plane (the ‘P-channel’) and fluorescence that is parallel to the excitation plane (the ‘S-channel’), and then it calculates FP in millipolarization units (mP) as follows: mP = [(S – P * G) */* (S + P * G)] * 1000. The ‘G-factor’ is measured by the instrument as a correction for any bias toward the P channel (16). Polarization values of each ALEXA647-labeled ASO in 1× PBS at 2 nM concentration were subtracted from each measurement. *K*_d_ values were calculated with GraphPad Prism 5 software (GraphPad Software, La Jolla, CA, USA) using non-linear regression for curve fit assuming one binding site.

Size exclusion chromatography was performed on a Zenix C 300 column (Sepax, Newark, DE) in PBS utilizing UV and β-RAM detection. The radio-labeled ASO was synthesized by mixing of 5′-hexylamino modified ASO dissolved in 0.1 M sodium tetraborate, pH 8.5 and Bolton-Hunter I-125 reagent (2 mC_i_, PerkinElmer, Waltham, MA) dissolved in DMSO for 2 h. The reaction was quenched with 2 M aq. NaOH, the conjugate was purified using a Sephadex G-25 NAP column and concentrated to 0.5 mL by applying a stream of nitrogen to the vial. The ASO was incubated in 50% EDTA treated plasma from the different investigated species before SEC analysis for at least 1 h at room temperature. Mice lacking HRG were obtained from the RWTH Aachen (Germany).

Affinity chromatography and proteomics analysis were applied to identify plasma proteins that interact with ASOs by coupling hexylamine conjugated ASOs to Sepharose with carboxyl groups activated as N-hydroxy succinimide (NHS) esters (17,18). Resigns with four different ASO designs (5–10–5 MOE PS, DNA PS, 5–10–5 MOE PO/PS and 3–10–3 cEt PS) were packed into columns. Human plasma was injected onto columns and increasing amount of salt of 200, 750 mM and 2 M ammonium acetate was used to elute proteins bound to the ASO-conjugated resign. The proteins of the 750 mM elution of each ASO design was analyzed utilizing shotgun proteomic by MS Bioworks (Ann Arbor, MI, USA). No proteins were eluted from a column with a 5–10–5 MOE PO coupled resign, demonstrating identified proteins were specific to utilized ASO designs.

C57BL/6 mice were treated twice a week for two weeks with nontargeting control 5–10–5 MOE PS ASO with the sequence CCTTCCCTGAAGGTTCCTCC or HRG-targeted 5–10–5 MOE PS ASO TTATTCAGAATATTGTCCTC at 50 mg/kg. Six days after the last administration, mice were treated with a single administration of active ASO targeting SRB 3–10–3 cEt ASO TCAGTCATGACTTC or the 5–10–5 MOE SRB ASO with the sequence GCTTCAGTCATGACTTCCTT at doses ranging from 0.5 mg/kg to 100 mg/kg. Two days after administration of PTEN or SRB-1 ASOs, animals were terminated, and plasma and liver samples were collected according to approved protocols. Treatment with HRG ASO reduced HRG protein by at least 80% measured by ELISA.

Animal experiments were conducted in accordance with the American Association for the Accreditation of Laboratory Animal Care guidelines and were approved by the Animal Welfare Committee (Cold Spring Harbor Laboratory's Institutional Animal Care and Use Committee guidelines). The animals were housed in microisolator cages on a constant 12-h light–dark cycle with controlled temperature and humidity and were given access to food and water *ad libitum*. Tissues were collected, weighed, flash frozen on liquid nitrogen, and stored at −60°C.

## RESULTS

### Development of fluorescence polarization binding assay

**Figure 1.**
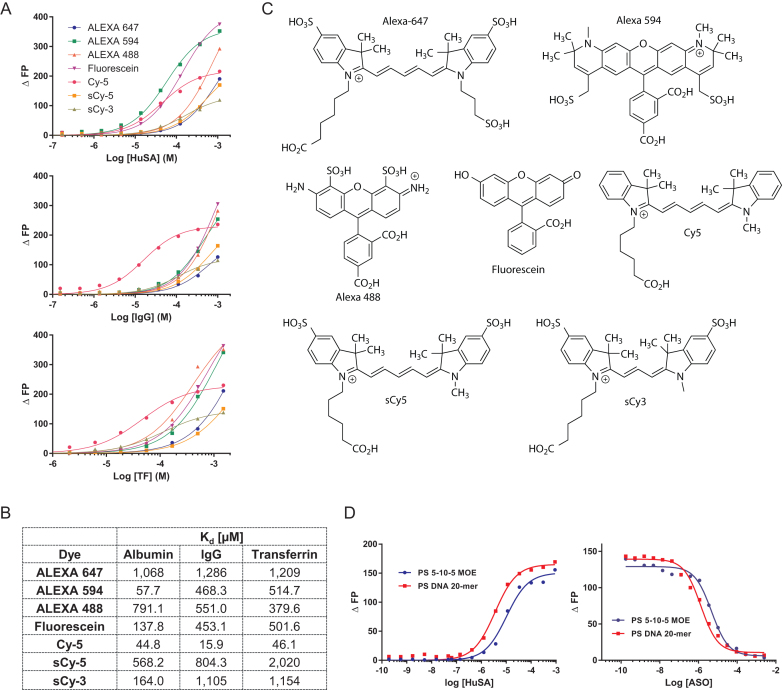
Identifying the optimal dye for measuring interactions of ASOs with plasma proteins. (**A**) Dose–response curves and (**B**) Binding constants of seven commonly used fluorescent dyes to human serum albumin, IgG and transferrin. (**C**) Structures of fluorescent dyes. (**D**) Comparison of direct binding with competitive binding of ALEXA 647-labelled ASOs with human serum albumin. ASO sequence CTGCTAGCCTCTGGATTTGA. Dose response curves were calculated using GrapPad Prism 5 software.

ASOs were fluorescently labeled for use in FP assays. In order to measure weak interactions between ASOs and proteins, the contribution of the fluorescent dye to the binding must be minimal. Therefore, we measured the binding constants of several commercially available dyes to human albumin, IgG, and transferrin (Figure 1A, B). All dyes were measures in their carboxylated form (Figure 1C). For Alexa Fluor 647, sCy5 and Alexa Fluor 488 weak binding to all investigated proteins was observed, while Cy5 had the tightest binding. Alexa Fluor 549 and fluorescein bound relative tightly to albumin, but with lower affinity to IgG and transferrin. Since Alexa Fluor 647 had the weakest binding in the millimolar range to two of the three investigated proteins, ASOs utilized in this study were labeled with this dye. Next, we evaluated if binding measured directly or by competition give comparable results. Binding constants (*K*_d_) of some representative full PS ASOs labeled with Alexa Fluor 647 to human serum albumin in phosphate buffered saline (PBS) were determined at pH 7.4. The binding constants of the ICAM ASO and PTEN MOE gapmer were 4.5 and 2 μM respectively (Figure 1D). Almost identical results with inhibition constants (*K*_i_) of 6 and 2 μM for the two ASOs were determined when the Alexa Fluor 647 labeled ASOs were competed with unlabeled ASO (Figure 1D). The concordance between the *K*_d_ and *K*_i_ values confirmed minimal contribution of the Alexa Fluor-647 dye to protein binding.

### Binding of ASOs to albumin

**Figure 2.**
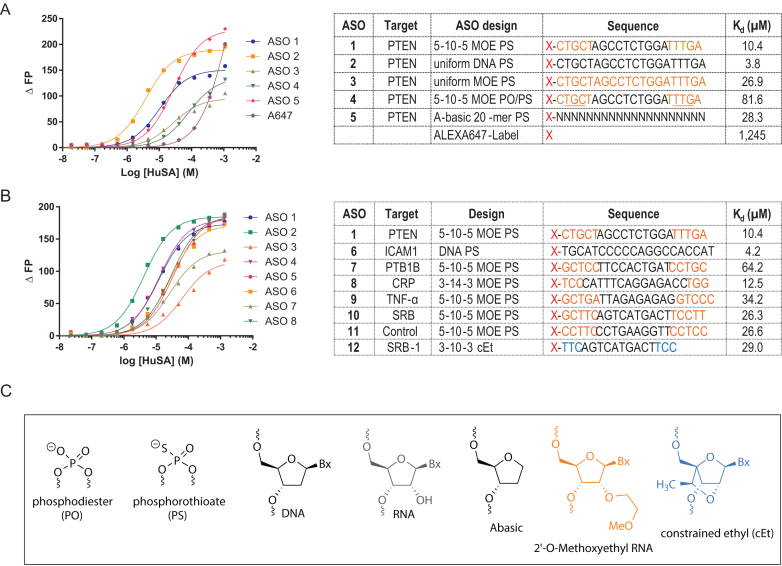
Effect of ASO design and sequence on binding of ASOs to human serum albumin. (**A**) Dose-response curves and dissociation constants of ASOs with varying PS and MOE content. (**B**) Dose-response curves and dissociation constants of ASOs with varying sequences and chemical designs. (**C**) Structures of ASO chemical modifications used in this study. Orange letters indicate MOE, Black DNA, Blue cEt, N = PS modified Abasic oligonucleotides, underlined letters represents PO linkage, X = Alexa 647 dye.

Serum albumin is the most abundant plasma protein and binding constants to PS ASOs in the micro molar range have been reported previously (19,20). We utilized an ASO targeting PTEN mRNA (21) with different sugar and backbone designs to evaluate how structural changes in the ASO affect binding to albumin (Figure 2C). For the MOE gapmers with the full PS-backbone (5–10–5 MOE PS) a binding constant of 10.4 μM was determined. A full DNA version of the PTEN ASO (DNA PS) bound 3 times tighter, while the binding of the full MOE version (full MOE PS) was 2.5 times weaker (Figure 2A). We also tested a mixed backbone gapmer motif, where 4 PS linkages were substituted with PO linkages in the MOE wings (5–10–5 MOE PO/PS) and observed an 8-fold drop in binding compared to the 5–10–5 MOE PS. Even a 20-mer PS oligomer with no nucleobases (abasic) bound albumin with a 3-fold reduced affinity compared to the 5–10–5 MOE PS ASO. These data emphasized the importance of the phosphorothioate linkage to albumin binding and suggest only limited contribution from the nucleobases to the interaction.

We next evaluated the effect of sequence on binding to albumin. For a set of PS ASOs with different sequences comparable binding was observed (Figure 2B). The first-generation ICAM DNA sequence had the tightest binding followed by a series of MOE gapmer ASOs and the 3–10–3 cEt ASO. For the 5–10–5 MOE gapmers (PTEN, SRB, PTB1B, TNF-α and control ASO) a range of binding constants from 10.4 μM for the PTEN to 64.2 μM for the PTB1B ASO were observed, suggesting small contributions from the nucleobases.

### Binding of ASOs to the 20 most abundant proteins in human plasma

**Figure 3.**
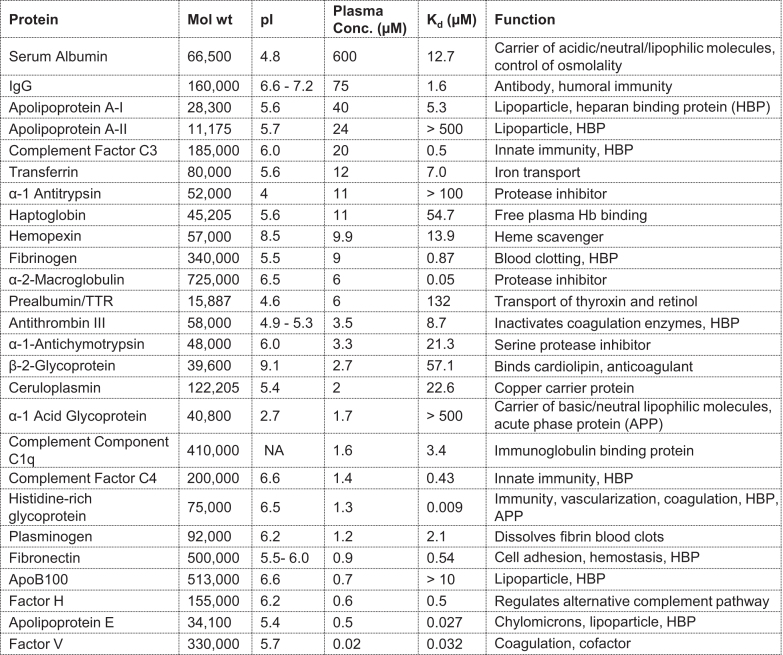
Characterizing the binding of an Alexa 647 labelled 5–10–5 PS MOE ASO (X-CTGCTAGCCTCTGGATTTGA) with 20 most abundant plasma proteins. Protein, molecular weight, *iso*-electric point (pI), concentration in plasma, ASO dissociation constant, and biological function of the protein. Binding curves are provided in the supplement information (Supplementary Figure S1)

We chose the relative abundance of proteins in human plasma and commercial availability of proteins as the criteria for evaluation in the binding assay (22,23). All chosen proteins were evaluated for binding to the PTEN 5–10–5 MOE PS ASO (Figure 3). The tightest binding constants were observed for α-2-macroglobulin (A2M) (23), coagulation factor V (24), apolipoprotein E (25), and histidine-rich glycoprotein (HRG) (26), with binding constants in the low nM range (50, 32, 27 and 9 nM respectively). For fibrinogen (27), coagulation factor H (28), complement factor C3 and C4 (29), and fibronectin (30), binding constants of several hundred nM were determined (870, 500, 500, 430 and 540 nM respectively). Plasma proteins such as antithrombin III (31), transferrin (32), apolipoprotein AI (33), plasminogen (34), complement component C1q (35), and IgG (36), bound the PTEN ASO in the low μM range (8.7, 7.0, 5.3, 2.1, 3.4 and 1.6 μM respectively). β-2-glycoprotein (37), haptoglobin (38), ceruloplasmin (39), α-1-antichymotrypsin (40) and hemopexin (41), revealed binding constants comparable or slightly weaker compared to serum albumin (57.1, 54.7, 22.6, 21.3 and 13.9 μM respectively). A group of plasma proteins with weak or no observed binding include prealbumin (42), α-1 acid glycoprotein (AGP) (43), apolipoprotein AII (44), and α-1-antithrypsin (45). Apolipoprotein B100 (46) did not show binding at the highest tested concentration of 5 μM. The range of determined binding constants from low nM to high μM demonstrates that many factors influence the interactions of PS ASOs with plasma proteins.

### Influence of ASO chemistry, design, and flexibility on interactions with plasma proteins

**Figure 4.**
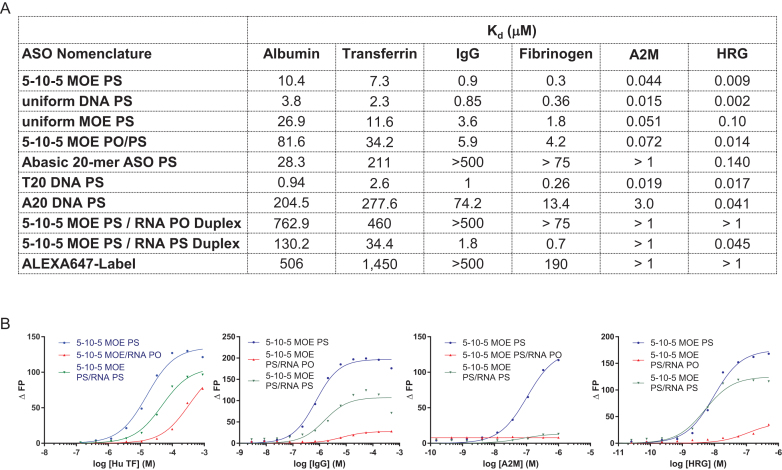
Characterizing the effect of ASO design, modification pattern, sequence and single-strandedness for select plasma proteins. (**A**) Binding constants of different ASO designs for selected human plasma proteins. (**B**) Binding curves showing differences between binding of single-stranded and double-stranded 5–10–5 MOE PS ASOs to select plasma proteins. Complementary RNA used for duplexation was either unmodified or fully PS-modified. ASO sequence 5′-CTGCTAGCCTCTGGATTTGA. RNA complement 5′-UCAAAUCCAGAGGCUAGCAG (unmodified) or 5′-UCAAAUCCAGAGGCUAGCAG (PS-modified).

We next investigated the effect of ASO design and chemistry on interactions of PS ASOs with a panel of plasma proteins including albumin. The rank order of binding for the 4 different PTEN ASO motifs to transferrin, IgG, fibrinogen, A2M and HRG was identical to that observed for binding to albumin (Figure 4A). The full DNA PS was the tightest binding motif, followed by the 5–10–5 MOE PS and the full MOE PS. For all proteins, the 5–10–5 MOE PO/PS exhibit the weakest binding in the series. Interestingly the relative binding differences for the different PTEN ASO motifs become smaller when the binding constants become tighter. For albumin, the full DNA PS binds 25-fold tighter than the 5–10–5 MOE PO/PS, for transferrin this ratio is 15, for fibrinogen 5 and A2M only 2.

As described above, albumin had a 3-fold weaker binding constant to a 20-mer PS abasic oligomer compared to the PTEN 5–10–5 MOE PS, underscoring the significance of the phosphorothioate linkage and lack of contribution from the nucleobases to protein binding. In contrast, binding of the abasic oligomer dropped 70-fold for transferrin, 500-fold for IgG, 225-fold for fibrinogen and 25-fold for A2M as compared to the PTEN MOE PS ASO. The effect was less pronounced for HRG, where the binding dropped 10-fold. Thus, nucleobases contribute much more to the affinity for these proteins.

To investigate the influence of ASO strand-flexibility on plasma protein binding we measured the binding of a 20-mer thymidine or adenine homo-polymeric DNA PS oligonucleotides (dT20 and dA20) to our key set of plasma proteins. The two homo-polymeric sequences can be regarded as a model for sequences with no (dT20) or maximum (dA20) base self-stacking properties (47). In all cases, the binding to dT20 was tighter compared to dA20. The extent of the binding difference was significant for most proteins (100-fold for transferrin, 75-fold for IgG) but only 3-fold for the tight binding HRG.

Next, we explored how duplexed ASOs change the binding to key plasma proteins. For the PTEN ASO duplexed with a phosphodiester (PO) RNA, all the investigated proteins showed a substantial loss of binding affinity (Figure 4A and B). Interestingly, even the PTEN ASO duplexes with a PS RNA exhibited a significantly lower binding affinity for all proteins except HRG. This argues that a flexible ASO structure facilitates efficient binding to these major plasma proteins.

### Effect of pH and salt on interaction with key plasma proteins

**Figure 5.**
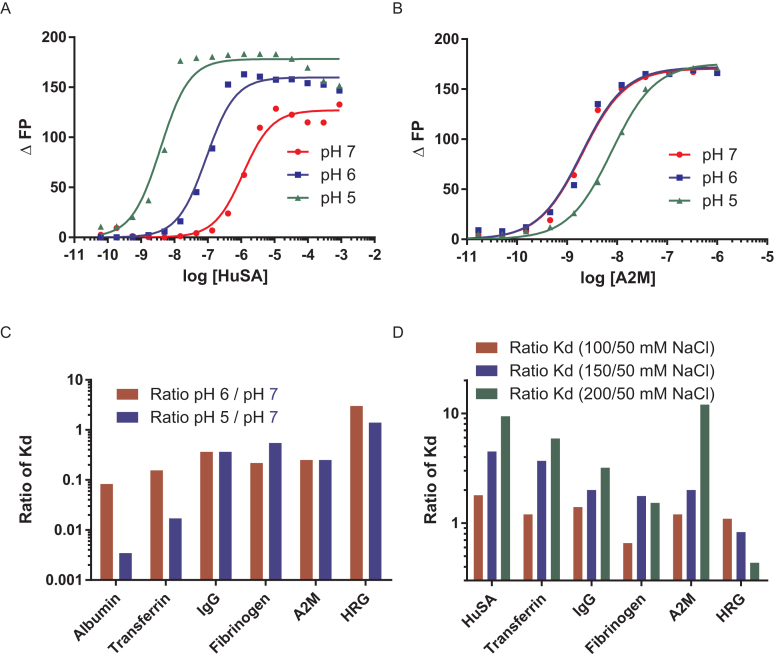
Characterizing the effect of pH and salt concentration on the interactions of PS ASOs with plasma proteins. Binding of ASO to (**A**) human serum albumin is affected while binding of (**B**) A2M is minimally affected by pH changes. (**C**) Ratio of binding constants at pH 5 and 6 compared to pH 7 for 6 proteins (D) Ratio of binding constants at 100, 150 and 200 mM NaCl buffer salt concentration compared to binding at 50 mM NaCl salt concentration for six proteins. ASO sequence and design PS 5–10–5 MOE ASO X-CTGCTAGCCTCTGGATTTGA.

To investigate the contribution of electrostatic interactions on ASO protein binding, we measured the binding of the 5–10–5 MOE PTEN PS to a key set of plasma serum proteins (albumin, transferrin, IgG, fibrinogen, A2M and HRG) utilizing a 5 mM phosphate buffer with 150 mM NaCl at pH 7, 6 and 5. The binding for albumin (Figure 5A) and transferrin increased ∼10-fold for each pH unit. This observation can be rationalized by increasing the positive charge on the protein through protonation of amino acids like histidine, and deprotonation of acidic amino acids. A significantly smaller (<3-fold) effect of pH on the binding constant of the ASO was observed for IgG, fibrinogen, A2M (Figure 5B) or HRG. Plotting the ratio of the binding constants at pH 5 and 6 compared to pH 7 clearly demonstrates this difference (Figure 5C).

A similar trend was observed when we investigated the salt dependence of the binding of these proteins to the PTEN MOE PS ASO (Figure 5D). Increasing the sodium chloride concentration from 50 to 200 mM in the buffer decreased the binding for albumin and A2G 10-fold, 5-fold for transferrin and 2-fold for IgG. Binding to fibrinogen was only marginally affected by salt and an increase in binding was observed for HRG at higher salt concentrations.

### Interactions of ASOs with human, mouse, rat and monkey albumin and plasma

**Figure 6.**
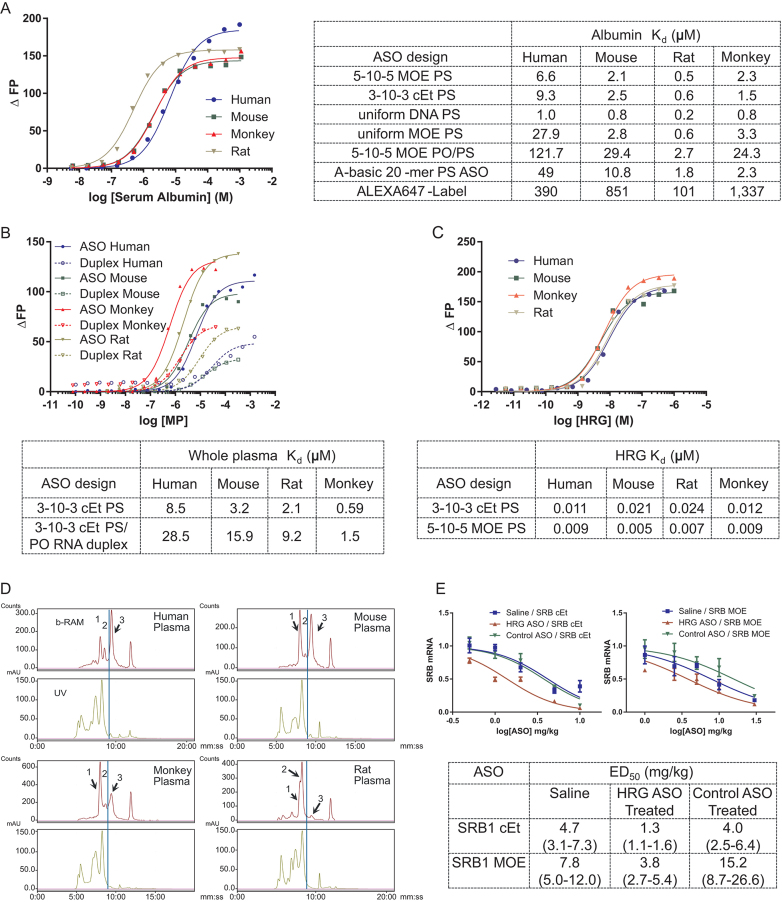
Characterizing the interactions of ASOs with plasma proteins from different species. (**A**) Effect of ASO design and modification on binding to human, mouse, rat, and monkey serum albumin. (**B**) Binding of MALAT-1 3–10–3 cEt ASO and corresponding RNA PO duplex to human, mouse, rat and monkey plasma. (**C**) Dissociation constants for human, mouse, rat, and monkey HRG to MALAT-1 3–10–3 PS and PTEN 5–10–5 MOE PS ASOs. Depicted is the binding of the PTEN PS ASO to HRG from the investigated species. (**D**) Size exclusion chromatography binding profile of a 125I-labelled 5–10–5 MOE PS ASO to plasma from human, mouse, monkey, and rat. 1 indicates ASO elutes bound to HRG, 2 indicates ASO elutes bound to serum albumin and 3 indicates unbound ASO. (**E**) Knock-down of HRG increases potency of a 3–10–3 cEt and a 5–10–5 MOE SRB ASO. SRB knockdown was evaluated after HRG was knocked down 90% utilizing a HRG ASO. In a separate group a nontargeting control ASO was used with no HRG knockdown.

We next examined interactions of PS ASOs with plasma proteins from man, monkey, mouse, and rat, which represent the most commonly used species for the preclinical assessments of nucleic acid-based therapeutics. We first measured binding constants of a series of PTEN ASOs to human, mouse, rat, and monkey albumin (Figure 6A). Rat albumin had the tightest binding constant of 500 nM for the 5–10–5 MOE PS followed by monkey and mouse with dissociation constants of 2.1 and 2.6 μM, respectively. We determined a binding constant of 6.6 μM for human serum albumin, the weakest affinity compared to other species and comparable to the result reported above for binding to albumin utilizing a different commercial source. The different PTEN sequence motifs demonstrated a similar binding behavior for all four species with the tightest binding observed for the full DNA PS, followed by the 5–10–5 MOE PS, the full MOE and the 5–10–5 MOE PO/PS. We also examined the interaction of the full PS ASOs with HRG from different species and found that all of them bind PS ASOs with equally high affinity (Figure 6C).

To determine if measuring interactions with individual proteins is relevant to interactions in whole plasma, we measured the binding affinity of PS ASOs to whole plasma from rat, mouse, man, and monkey (Figure 6B). We found that measuring interactions with individual proteins can indeed be relevant as we observed the tightest binding to monkey plasma which has higher levels of HRG relative to other species. Rat plasma also exhibited increased binding given the higher affinity of rat albumin for PS ASOs.

To determine if the binding measured by individual proteins can be reassessed within a biological matrix, size exclusion chromatography (SEC) was performed with a 5–10–5 MOE SRB PS ASO incubated into human, mouse, rat, and monkey plasma at 5 μM concentration. Binding to individual fractions was monitored by spiking the parent ASO with a small portion of ^125^I-labelled ASO with the same sequence as the parent. The profiles display a broad distribution of the SRB ASO to many proteins with a few distinct peaks visible, which are consistent with binding to albumin, HRG, and unbound ASO (Figure 6D). We confirmed the HRG binding peak in the wildtype mouse plasma by comparing the profile to plasma from a HRG knockout mouse (48), where no HRG ASO binding peak was present (Supplementary Figure S2).

The relative peak height of the HRG binding peak in the different species correlates with the protein level in these species, with human having the lowest level of approximately 1 μM, followed by rat and mouse with 3 μM and monkey with 5 μM. For rat, the highest albumin binding was observed consistent with the tight binding measured for the isolated protein. Consequently, the smallest unbound peak was observed for rat, followed by monkey, mouse and human. A similar rank order was observed in the evaluation of species differences in plasma binding of a first generation ASO utilizing a filter binding assay. (12) When evaluating these profiles, it must be considered that weaker ASO protein interaction dissociate during the separation, which explains the rather small albumin binding peak.

The relevance of HRG-binding on ASO activity was confirmed by knocking down HRG in mice using an ASO, followed by administration of ASOs targeting SRB1 mRNA (49). Transiently reducing HRG in the mouse resulted in a 2-fold enhancement in activity for the SRB1-ASOs (Figure 6E) suggesting that tight binding to HRG shunts the ASO into less productive tissue compartments.

### Identification of proteins which interact with ASOs in human plasma

**Figure 7.**
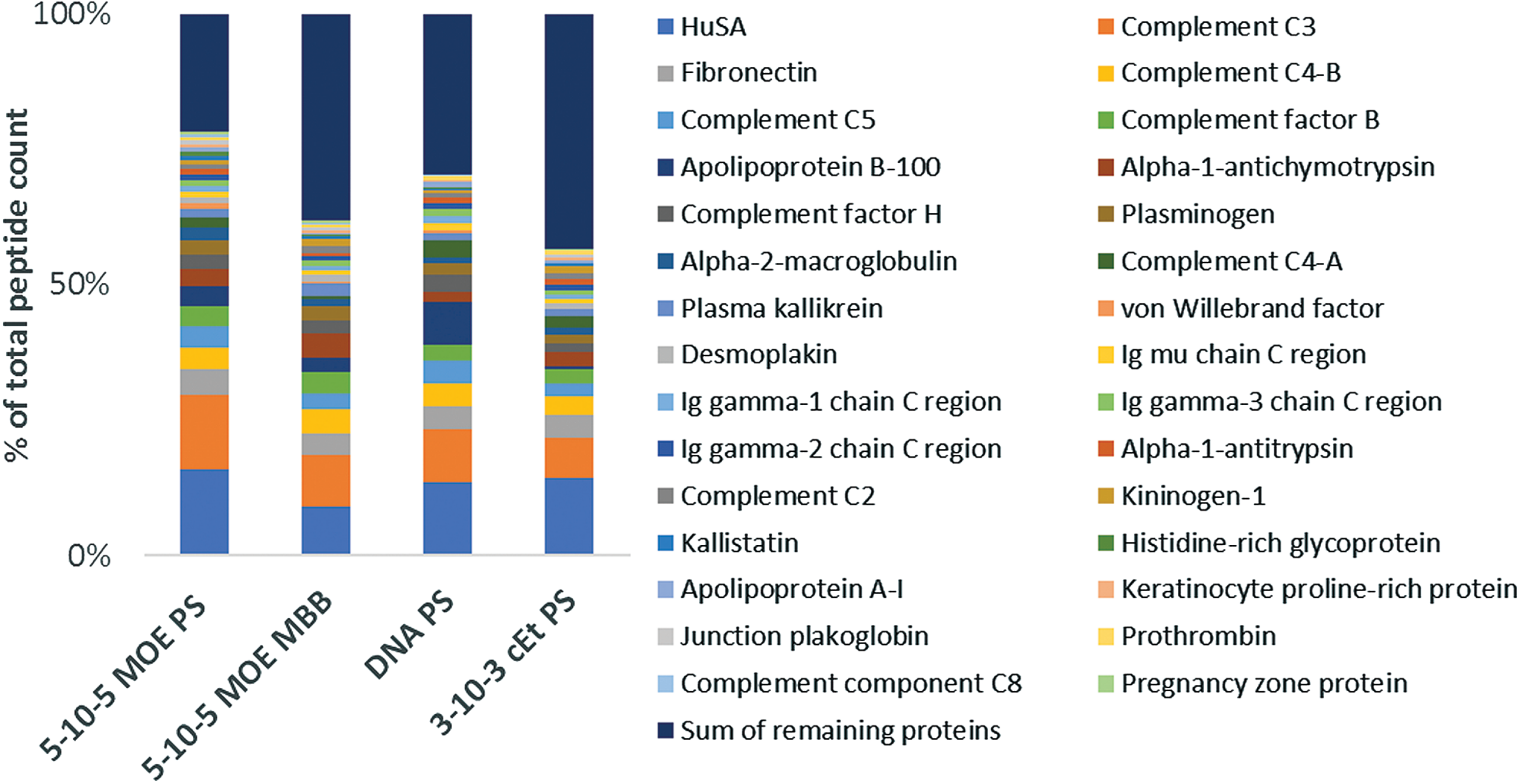
List of identified human plasma proteins binding to different ASO designs and relative abundance based on peptide counts (only the 30 most abundant proteins are shown, full list is tabulated in the supplement information, Supplementary Table S1).

We utilized ASO affinity chromatography and proteomics to identify plasma proteins that interact with ASOs by adapting a procedure described in the literature for identifying heparin binding proteins (17,18). Four different ASO designs (5–10–5 MOE PS, DNA PS, 5–10–5 MOE PO/PS and 3–10–3 cEt PS) were investigated to evaluate the influence of different sugar and backbone modifications. ASOs modified with a hexylamino group at the 5′-position were conjugated to Sepharose with carboxyl groups activated as *N*-hydroxy succinimide (NHS) esters. Human plasma was run through the columns and the proteins were eluted by increasing the concentration of ammonium acetate in the eluent buffer. Proteins collected from the 750 mM ammonium acetate salt elution were identified by shotgun proteomics with relative protein quantitation performed by peptide counts. Using a 5–10–5 MOE PS ASO as the capture ligand, albumin was the most abundant protein by peptide counts, followed by complement C3, fibronectin, complement C4, complement C5 and complement factor B (Figure 7). Peptide counts can only be regarded as a semiquantitative measure to evaluate the most abundant proteins in the assay due to biases based on protein size, relative abundance, and peptide sequence (50,51).

A total of >200 proteins were identified from the salt elution, confirming that the PTEN ASOs bind many plasma proteins (Supplementary Table S1). Many of the proteins identified with high peptide counts have been classified as tight binding proteins with binding constants in the nM range determined by the FP binding assay. Examples of these proteins are complement C3, complement C4, fibrinogen, complement factor H and A2M. Consistent with the classification as weak binding proteins, transferrin, haptoglobin, hemopexin, prealbumin and α-1 acid glycoprotein, were not present amongst the 30 proteins with the most peptide counts as listed in Figure 7, but still could be identified in the salt elution with rather low counts (Supplementary Table S1). Not surprisingly, HRG was identified as a binding protein but with rather low peptide counts given its high affinity to ASOs measured by FP. Given that the binding of HRG to ASOs is minimally affected by salt, this presumably makes it difficult to elute HRG from the column-linked ASO. Besides complement C3 and C4, for which we determined binding constants in the nM range, several other complement proteins (C2, C5 and C8) were identified by affinity chromatography with high peptide count indicating that they have considerable affinity for 5–10–5 MOE PS ASO.

A very similar rank order of identified proteins was obtained when the other ASO design (DNA PS, 5–10–5 MOE PO/PS and 3–10–3 cEt PS) were tested as affinity chromatography media. Albumin was identified as the protein with the highest peptide count, followed by the same high affinity proteins as described for the 5–10–5 MOE PS ASO. The most obvious difference was the higher rank order for some immunoglobulins from the uniform DNA PS design. Otherwise the differences are rather subtle, even for the ASO with the mixed backbone (PO/PS) chemistry, which showed much lower binding affinity to many isolated proteins compared to 5–10–5 MOE PS or 3–10–3 cEt PS ASO.

## DISCUSSION

Unmodified nucleic acids are negatively charged macromolecules which are rapidly degraded in plasma and/or eliminated in the urine after injection into an animal. Chemical modifications to the backbone and sugar moieties have been employed to enhance stability from nuclease-mediated degradation. When designed appropriately, these modifications can enhance mechanistic potency and metabolic stability which improves antisense activity and permits less frequent dosing in the clinic. In addition, chemical modifications like the PS-backbone enhances association with plasma proteins which helps reduce filtration in the kidney and facilitates ASO distribution from the site of injection to peripheral cells and tissues in animals. Understanding the nature of interactions of PS ASOs with individual plasma proteins and the chemical features on the ASO which modulate these interactions, is important to address the broader question about how nucleic acid drugs distribute to tissues and achieve cellular entry. In this study, we report our initial characterizations of the interactions of PS ASOs with plasma proteins using an FP-based assay.

The number of PS linkages and single stranded nature of the ASO appeared to be the most important determinants for interaction with proteins with contributions from the nucleobases for proteins such as transferrin and IgG but not for albumin. Single stranded PS-DNA appeared to be the strongest binder while 2′-modifications such as MOE and cEt had modest effects for further modulating these interactions. Mixed backbone (MBB) designs which combine PS in the DNA gap and PO linkages in the MOE wings showed reduced binding for all proteins evaluated. Unconjugated MBB ASOs have sufficient plasma protein binding to facilitate distribution from the site of injection. However, these designs show reduced activity as lowering PS content reduces affinity for cell-surface proteins which facilitate ASO entry into cells (52). To address this, MBB ASO designs have recently been evaluated as GalNAc-conjugates (53) which enhance association of the ASO with a cell-surface receptor (ASGR). A MBB 5–10–5 MOE GalNAc-ASO targeting lipoprotein A recently showed ∼30-fold enhanced potency and improved tolerability relative to the parent full PS ASO in the clinic (54).

The rigidity of the nucleic acid also appeared to have an impact as a PS dA 20-mer showed 100-fold reduced affinity as compared to a PS dT 20-mer. Polythymidylate oligonucleotides were recently reported to have more strand flexibility because of reduced intra-strand stacking interactions of the nucleobases (55). Similarly, ASO/RNA duplexes which are more rigid with well-defined helical geometries, showed significantly reduced protein binding suggesting that the flexibility imparted by the single stranded nucleic acid facilitates interactions with proteins. Interestingly, ASO/RNA duplexes also showed reduced binding affinity to whole plasma as compared to the single stranded ASOs. These observations provide a rationale for why nucleic acid therapeutics such as siRNA or even the recently reported ASO duplexes are often modified using hydrophobic moieties such as cholesterol or tocopherol to provide a handle for interactions with plasma lipoproteins to facilitate tissue distribution and cellular entry (56–59).

Electrostatic interactions play a significant role in binding to most of the investigated proteins as revealed by the salt dependence studies. Of the weaker binding proteins, salt concentration had the biggest effect on albumin, while IgG binding was less affected by salt. Of the tighter binding proteins, salt concentration had the most pronounced effect on A2M affinity, whereas HRG binding increased slightly with higher salt. A similar result was observed for the pH dependence studies. Albumin showed the most pronounced increase in binding at lower pH, while IgG was less affected. HRG had almost no change in binding at lower pH, while A2M and fibrinogen showed a modest increase in affinity. These results are in contrast to the interactions of PS ASOs with the stabilin receptors where binding was reduced at lower pH (4). Several professional endocytic receptors like the stabilins, TfR and ASGR have pH sensitive binding with their substrates to facilitate release of cargo within endosomal compartments thereby permitting recycling of the receptor back to the cell-surface. Thus, pH dependency of ASO-protein interactions could be relevant as ASOs internalized as complexes with certain plasma proteins can be trapped within endosomal compartments where the pH is lower than in plasma.

PS ASOs bind to many plasma proteins with a wide range of affinities. PS ASOs bind to more abundant plasma proteins such as albumin, IgG, transferrin, apolipoprotein A and complement C3 with sub to low micromolar dissociation constants. In contrast, PS ASOs bind to some less abundant plasma proteins such as HRG, A2M, factor V and apolipoprotein E with 5–50 nanomolar binding constants. In contrast, proteins such as α-1 acid glycoprotein, α-1 antitrypsin, prealbumin (TTR) show minimal binding to PS ASOs. While an exhaustive analysis of the interactions of PS ASOs with plasma proteins was beyond the scope of the current study, we found that PS ASOs showed 2-fold improved activity in mice upon knockdown of plasma HRG. Similarly, PS ASOs also exhibit 2-fold improved activity in A2M knockout mice (10) suggesting that very tight interactions with plasma proteins can hinder activity by shunting the ASO into less productive uptake pathways and/or tissue compartments.

Interactions with specific plasma proteins can potentially modulate the tissue distribution of PS ASOs and could be adopted as a strategy to enhance ASO potency in tissues beyond the liver. As an example, albumin is actively transported across the capillary endothelium via caveolin-1 mediated transcytosis (60) and almost 60% of the total albumin resides in the interstitial space in tissues such as skeletal muscle, fat and the skin (61). Unlike the liver, where sinusoidal capillaries allow macromolecular therapeutics such as ASOs to exit the vasculature and enter the tissue interstitium, tissues such as muscle have an intact endothelium and basement membrane which must be traversed to gain access to the interstitium and the cell surface of the parenchymal cells of the tissue. Thus, tighter but reversible binding to plasma proteins such albumin could enhance ASO uptake in the muscle by facilitating transcytosis across the capillary endothelium.

Significant species-specific differences in total plasma protein binding were observed utilizing SEC. Most pronounced were the differences for HRG, which was clearly visible as a distinct peak in the chromatograms and binds to all species with very high affinity. This effect was perhaps most striking for monkey plasma which has the highest concentration of plasma HRG (5 uM) amongst the species evaluated. This observation may be biologically relevant as PS ASOs typically show reduced potency in monkeys, which have high plasma HRG concentration, as compared to mice and man (62). The enhanced binding to monkey plasma proteins was further confirmed by measuring the aggregate binding affinity of PS ASOs to plasma from man, rat, mouse, and monkey. These observations provide a potential rationale for why PS ASOs show reduced activity in the monkey as compared to mouse and man.

In summary, we demonstrated that PS ASOs interact with many different plasma proteins with a wide range of binding affinities. Distinct differences in pH- and salt dependency along with a pronounced preference for single stranded oligonucleotides, demonstrates that several structural features of the ASO govern these interactions. The FP assay appears to be well suited to evaluate binding characteristics of PS ASOs to proteins and may contribute to better understanding of these interactions and their significance in pharmacodynamic, pharmacokinetic and toxicity of nucleic acid therapeutics.

## Supplementary Material

Supplementary Data
