# Supplementary material for: Characterization of the interactions of chemically-modified therapeutic nucleic acids with plasma proteins using a fluorescence polarization assay
Source: Nucleic Acids Res. 2018 Dec 19;47(3):1110–22. doi: 10.1093/nar/gky1260 (PMC6379706; doi:10.1093/nar/gky1260)
Supplement: Supplementary Data [file gky1260_supplemental_files.pdf]

Supplementary Data

Figure S1: Binding of the 5-10-5 MOE PS PTEN ASO to the 25 most abundant human plasma proteins. Binding of serum albumin was included in each panel as a reference.

A

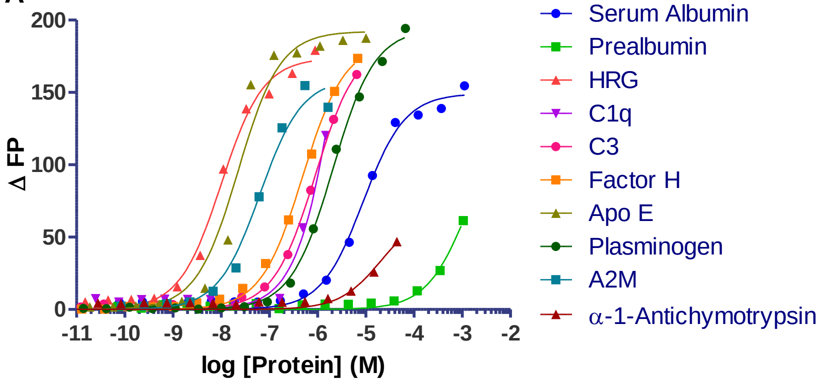

B

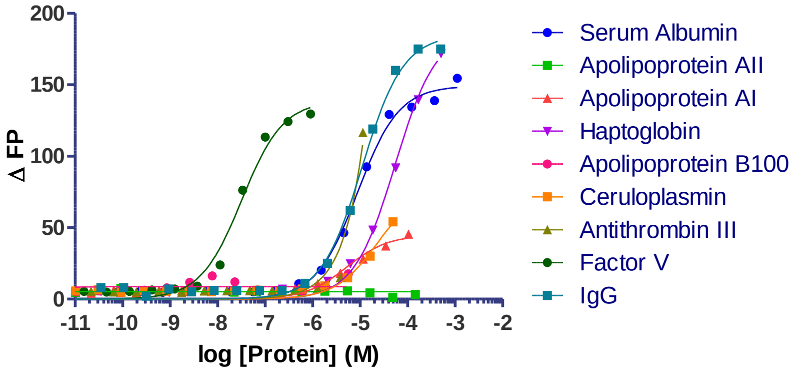

C

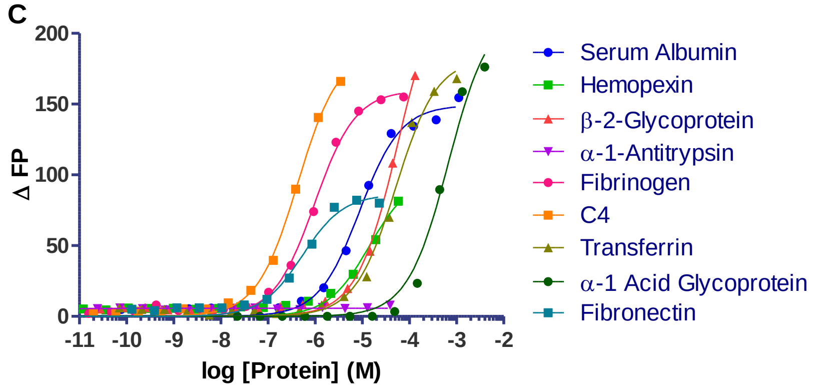

Figure S2: SEC binding profile of SRB MOE gapmer ASO to plasma from HRG -/- mice and wild type controls.

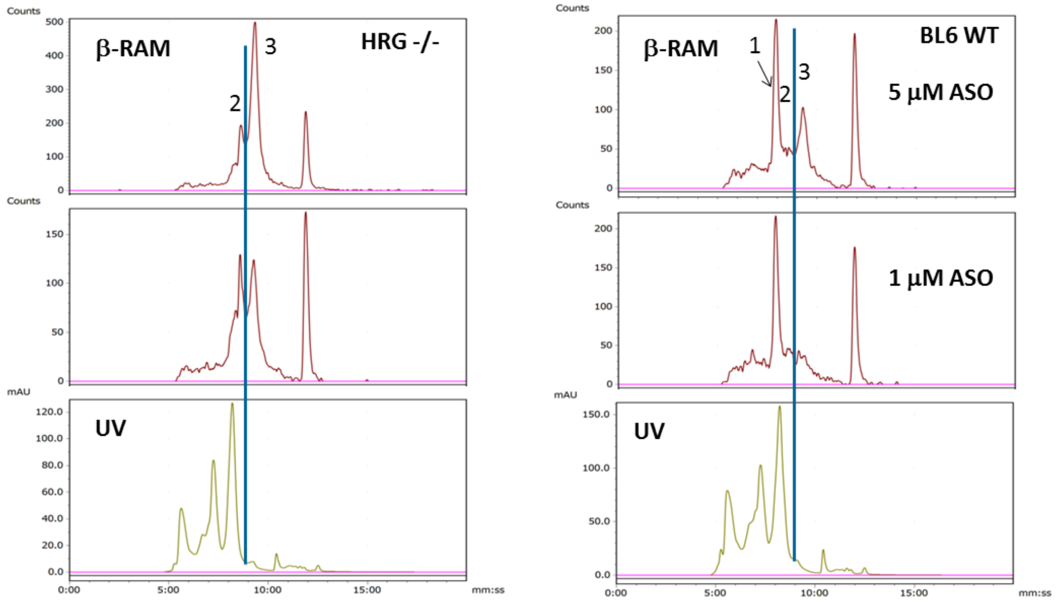

Table S1: Full list of identified plasma proteins binding to different ASO designs. Number of identified peptides listed for each protein and tested ASO design.

**Identified Proteins (243)**	**Accession Number**	**MW kDa**	**MOE PS**	**MOE MBB**	**DNA PS**	**cEt PS**
Serum albumin OS=Homo sapiens GN=ALB PE=1 SV=2	sp|P02768|ALBU_HUMAN	69	1691	900	2472	1811
Complement C3 OS=Homo sapiens GN=C3 PE=1 SV=2	sp|P01024|CO3_HUMAN	187	1470	951	1731	950
Fibronectin OS=Homo sapiens GN=FN1 PE=1 SV=4	sp|P02751|FINC_HUMAN	263	513	395	791	527
Complement C4-B OS=Homo sapiens GN=C4B PE=1 SV=2	sp|P0C0L5|CO4B_HUMAN	193	439	420	771	450
Complement C5 OS=Homo sapiens GN=C5 PE=1 SV=4	sp|P01031|CO5_HUMAN	188	403	289	741	299
Complement factor B OS=Homo sapiens GN=CFB PE=1 SV=2	sp|P00751|CFAB_HUMAN	86	401	410	545	337
Apolipoprotein B-100 OS=Homo sapiens GN=APOB PE=1 SV=2	sp|P04114|APOB_HUMAN	516	391	252	1433	68
Alpha-1-antichymotrypsin OS=Homo sapiens GN=SERPINA3 PE=1 SV=2	sp|P01011|AACT_HUMAN	48	356	457	362	322
Complement factor H OS=Homo sapiens GN=CFH PE=1 SV=4	sp|P08603|CFAH_HUMAN	139	289	234	529	211
Plasminogen OS=Homo sapiens GN=PLG PE=1 SV=2	sp|P00747|PLMN_HUMAN	91	261	245	389	195
Keratin, type I cytoskeletal 9 OS=Homo sapiens GN=KRT9 PE=1 SV=3	sp|P35527|K1C9_HUMAN	62	255	302	196	331
Alpha-2-macroglobulin OS=Homo sapiens GN=A2M PE=1 SV=3	sp|P01023|A2MG_HUMAN	163	252	151	208	181
Complement C4-A OS=Homo sapiens GN=C4A PE=1 SV=2	sp|P0C0L4|CO4A_HUMAN	193	213	56	593	255
Plasma kallikrein OS=Homo sapiens GN=KLKB1 PE=1 SV=1	sp|P03952|KLKB1_HUMAN	71	168	212	242	156
von Willebrand factor OS=Homo sapiens GN=VWF PE=1 SV=4	sp|P04275|VWF_HUMAN	309	120	42	61	12
Desmoplakin OS=Homo sapiens GN=DSP PE=1 SV=3	sp|P15924|DESP_HUMAN	332	113	114	38	143
Ig mu chain C region OS=Homo sapiens GN=IGHM PE=1 SV=3	sp|P01871|IGHM_HUMAN	49	113	96	207	81
Ig gamma-1 chain C region OS=Homo sapiens GN=IGHG1 PE=1 SV=1	sp|P01857|IGHG1_HUMAN	36	112	84	232	127
Ig gamma-3 chain C region OS=Homo sapiens GN=IGHG3 PE=1 SV=2	sp|P01860|IGHG3_HUMAN	41	110	89	241	91
Ig gamma-2 chain C region OS=Homo sapiens GN=IGHG2 PE=1 SV=2	sp|P01859|IGHG2_HUMAN	36	109	81	222	115
Alpha-1-antitrypsin OS=Homo sapiens GN=SERPINA1 PE=1 SV=3	sp|P01009|A1AT_HUMAN	47	100	54	175	146
Complement C2 OS=Homo sapiens GN=C2 PE=1 SV=2	sp|P06681|CO2_HUMAN	83	96	134	139	133
Kininogen-1 OS=Homo sapiens GN=KNG1 PE=1 SV=2	sp|P01042|KNG1_HUMAN	72	93	118	92	174
Kallistatin OS=Homo sapiens GN=SERPINA4 PE=1 SV=3	sp|P29622|KAIN_HUMAN	49	86	61	79	51
Histidine-rich glycoprotein OS=Homo sapiens GN=HRG PE=1 SV=1	sp|P04196|HRG_HUMAN	60	79	19	36	22
Apolipoprotein A-I OS=Homo sapiens GN=APOA1 PE=1 SV=1	sp|P02647|APOA1_HUMAN	31	76	34	199	63
Keratinocyte proline-rich protein OS=Homo sapiens GN=KPRP PE=1 SV=1	sp|Q5T749|KPRP_HUMAN	64	73	45	28	71
Junction plakoglobin OS=Homo sapiens GN=JUP PE=1 SV=3	sp|P14923|PLAK_HUMAN	82	73	56	21	59
Prothrombin OS=Homo sapiens GN=F2 PE=1 SV=2	sp|P00734|THRB_HUMAN	70	64	64	105	94
Complement component C8 beta chain OS=Homo sapiens GN=C8B PE=1 SV=3	sp|P07358|CO8B_HUMAN	67	61	19	68	5
Pregnancy zone protein OS=Homo sapiens GN=PZP PE=1 SV=4	sp|P20742|PZP_HUMAN	164	58	47	11	42
Extracellular matrix protein 1 OS=Homo sapiens GN=ECM1 PE=1 SV=2	sp|Q16610|ECM1_HUMAN	61	56	55	41	32
Plasma serine protease inhibitor OS=Homo sapiens GN=SERPINA5 PE=1 SV=3	sp|P05154|IPSP_HUMAN	46	55	116	64	72

Pigment epithelium-derived factor OS=Homo sapiens GN=SERPINF1 PE=1 SV=4	sp|P36955|PEDF_HUMAN	46	53	75	114	64
Ig alpha-1 chain C region OS=Homo sapiens GN=IGHA1 PE=1 SV=2	sp|P01876|IGHA1_HUMAN	38	53	50	102	71
Complement component C8 alpha chain OS=Homo sapiens GN=C8A PE=1 SV=2	sp|P07357|CO8A_HUMAN	65	53	46	96	47
Coagulation factor XII OS=Homo sapiens GN=F12 PE=1 SV=3	sp|P00748|FA12_HUMAN	68	50	92	89	70
Insulin-like growth factor-binding protein complex acid labile subunit OS=Homo sapiens GN=IGFALS PE=1 SV=1	sp|P35858|ALS_HUMAN	66	49	36	79	44
Ig kappa chain C region OS=Homo sapiens GN=IGKC PE=1 SV=1	sp|P01834|IGKC_HUMAN	12	48	37	109	40
Inter-alpha-trypsin inhibitor heavy chain H4 OS=Homo sapiens GN=ITIH4 PE=1 SV=4	sp|Q14624|ITIH4_HUMAN	103	44	160	213	189
Plasma protease C1 inhibitor OS=Homo sapiens GN=SERPING1 PE=1 SV=2	sp|P05155|IC1_HUMAN	55	44	141	110	166
Complement C1q subcomponent subunit B OS=Homo sapiens GN=C1QB PE=1 SV=3	sp|P02746|C1QB_HUMAN	27	43	19	44	13
Complement C1q subcomponent subunit C OS=Homo sapiens GN=C1QC PE=1 SV=3	sp|P02747|C1QC_HUMAN	26	42	23	36	13
Carboxypeptidase B2 OS=Homo sapiens GN=CPB2 PE=1 SV=2	sp|Q96IY4|CBPB2_HUMAN	48	40	45	58	16
Inter-alpha-trypsin inhibitor heavy chain H2 OS=Homo sapiens GN=ITIH2 PE=1 SV=2	sp|P19823|ITIH2_HUMAN	106	39	144	224	290
Fibrinogen alpha chain OS=Homo sapiens GN=FGA PE=1 SV=2	sp|P02671|FIBA_HUMAN	95	38	170	449	620
C4b-binding protein alpha chain OS=Homo sapiens GN=C4BPA PE=1 SV=2	sp|P04003|C4BPA_HUMAN	67	36	92	191	89
Hornerin OS=Homo sapiens GN=HRNR PE=1 SV=2	sp|Q86YZ3|HORN_HUMAN	282	35	69	19	69
Filaggrin-2 OS=Homo sapiens GN=FLG2 PE=1 SV=1	sp|Q5D862|FILA2_HUMAN	248	34	28	13	26
Serotransferrin OS=Homo sapiens GN=TF PE=1 SV=3	sp|P02787|TRFE_HUMAN	77	32	22	61	94
Immunoglobulin lambda-like polypeptide 5 OS=Homo sapiens GN=IGLL5 PE=2 SV=2	sp|B9A064|IGLL5_HUMAN	23	32	31	57	37
Inter-alpha-trypsin inhibitor heavy chain H1 OS=Homo sapiens GN=ITIH1 PE=1 SV=3	sp|P19827|ITIH1_HUMAN	101	29	51	111	184
Coagulation factor XI OS=Homo sapiens GN=F11 PE=1 SV=1	sp|P03951|FA11_HUMAN	70	29	8	29	13
Haptoglobin OS=Homo sapiens GN=HP PE=1 SV=1	sp|P00738|HPT_HUMAN	45	28	32	48	39
Beta-2-glycoprotein 1 OS=Homo sapiens GN=APOH PE=1 SV=3	sp|P02749|APOH_HUMAN	38	27	89	210	165
Protein S100-A8 OS=Homo sapiens GN=S100A8 PE=1 SV=1	sp|P05109|S10A8_HUMAN	11	26	40	11	22
Complement component C8 gamma chain OS=Homo sapiens GN=C8G PE=1 SV=3	sp|P07360|CO8G_HUMAN	22	26	15	39	19
Skin-specific protein 32 OS=Homo sapiens GN=XP32 PE=1 SV=1	sp|Q5T750|XP32_HUMAN	26	26	16	9	25
Sulfhydryl oxidase 1 OS=Homo sapiens GN=QSOX1 PE=1 SV=3	sp|O00391|QSOX1_HUMAN	83	26	12	12	5
Complement component C9 OS=Homo sapiens GN=C9 PE=1 SV=2	sp|P02748|CO9_HUMAN	63	25	95	161	137
Hemopexin OS=Homo sapiens GN=HPX PE=1 SV=2	sp|P02790|HEMO_HUMAN	52	25	2	101	67
Vitronectin OS=Homo sapiens GN=VTN PE=1 SV=1	sp|P04004|VTNC_HUMAN	54	24	57	76	62
Desmoglein-1 OS=Homo sapiens GN=DSG1 PE=1 SV=2	sp|Q02413|DSG1_HUMAN	114	23	29	5	27
Alpha-1-acid glycoprotein 1 OS=Homo sapiens GN=ORM1 PE=1 SV=1	sp|P02763|A1AG1_HUMAN	24	23	8	37	25
Immunoglobulin heavy variable 3-7 OS=Homo sapiens GN=IGHV3-7 PE=1 SV=2	sp|P01780|HV307_HUMAN	13	22	19	38	15
Afamin OS=Homo sapiens GN=AFM PE=1 SV=1	sp|P43652|AFAM_HUMAN	69	22	0	37	28
Ig lambda-2 chain C regions OS=Homo sapiens GN=IGLC2 PE=1 SV=1	sp|P0CG05|LAC2_HUMAN	11	22	17	64	29
Protein S100-A9 OS=Homo sapiens GN=S100A9 PE=1 SV=1	sp|P06702|S10A9_HUMAN	13	21	38	12	22

N-acetylmuramoyl-L-alanine amidase OS=Homo sapiens GN=PGLYRP2 PE=1 SV=1	sp|Q96PD5|PGRP2_HUMAN	62	20	33	53	37
Hepatocyte growth factor-like protein OS=Homo sapiens GN=MST1 PE=1 SV=2	sp|P26927|HGFL_HUMAN	80	20	20	6	2
Apolipoprotein E OS=Homo sapiens GN=APOE PE=1 SV=1	sp|P02649|APOE_HUMAN	36	19	26	54	16
Lysozyme C OS=Homo sapiens GN=LYZ PE=1 SV=1	sp|P61626|LYSC_HUMAN	17	19	27	11	11
Carboxypeptidase N subunit 2 OS=Homo sapiens GN=CPN2 PE=1 SV=3	sp|P22792|CPN2_HUMAN	61	19	30	23	32
Cornifin-A OS=Homo sapiens GN=SPRR1A PE=1 SV=2	sp|P35321|SPR1A_HUMAN	10	18	13	11	11
Proteoglycan 4 OS=Homo sapiens GN=PRG4 PE=1 SV=2	sp|Q92954|PRG4_HUMAN	151	17	19	26	15
Insulin-like growth factor-binding protein 3 OS=Homo sapiens GN=IGFBP3 PE=1 SV=2	sp|P17936|IBP3_HUMAN	32	17	38	10	10
Protein-glutamine gamma-glutamyltransferase K OS=Homo sapiens GN=TGM1 PE=1 SV=4	sp|P22735|TGM1_HUMAN	90	17	7	2	12
Alpha-1-acid glycoprotein 2 OS=Homo sapiens GN=ORM2 PE=1 SV=2	sp|P19652|A1AG2_HUMAN	24	17	14	9	16
Dermcidin OS=Homo sapiens GN=DCD PE=1 SV=2	sp|P81605|DCD_HUMAN	11	16	8	4	9
Complement factor H-related protein 1 OS=Homo sapiens GN=CFHR1 PE=1 SV=2	sp|Q03591|FHR1_HUMAN	38	15	31	19	18
Protein AMBP OS=Homo sapiens GN=AMBP PE=1 SV=1	sp|P02760|AMBP_HUMAN	39	14	62	47	97
CD5 antigen-like OS=Homo sapiens GN=CD5L PE=1 SV=1	sp|O43866|CD5L_HUMAN	38	14	25	26	17
Carboxypeptidase N catalytic chain OS=Homo sapiens GN=CPN1 PE=1 SV=1	sp|P15169|CBPN_HUMAN	52	14	9	24	13
Complement factor D OS=Homo sapiens GN=CFD PE=1 SV=5	sp|P00746|CFAD_HUMAN	27	13	5	10	4
Loricrin OS=Homo sapiens GN=LOR PE=1 SV=2	sp|P23490|LORI_HUMAN	26	13	7	2	3
Antithrombin-III OS=Homo sapiens GN=SERPINC1 PE=1 SV=1	sp|P01008|ANT3_HUMAN	53	12	10	49	40
Glyceraldehyde-3-phosphate dehydrogenase OS=Homo sapiens GN=GAPDH PE=1 SV=3	sp|P04406|G3P_HUMAN	36	12	24	7	16
Apolipoprotein D OS=Homo sapiens GN=APOD PE=1 SV=1	sp|P05090|APOD_HUMAN	21	12	9	17	17
Ficolin-2 OS=Homo sapiens GN=FCN2 PE=1 SV=2	sp|Q15485|FCN2_HUMAN	34	12	14	11	6
Complement factor H-related protein 2 OS=Homo sapiens GN=CFHR2 PE=1 SV=1	sp|P36980|FHR2_HUMAN	31	12	12	9	18
Haptoglobin-related protein OS=Homo sapiens GN=HPR PE=2 SV=2	sp|P00739|HPTR_HUMAN	39	12	11	21	13
Tetranectin OS=Homo sapiens GN=CLEC3B PE=1 SV=3	sp|P05452|TETN_HUMAN	23	11	10	14	18
Complement C1q subcomponent subunit A OS=Homo sapiens GN=C1QA PE=1 SV=2	sp|P02745|C1QA_HUMAN	26	11	5	11	3
Gelsolin OS=Homo sapiens GN=GSN PE=1 SV=1	sp|P06396|GELS_HUMAN	86	10	127	255	135
Clusterin OS=Homo sapiens GN=CLU PE=1 SV=1	sp|P10909|CLUS_HUMAN	52	10	15	32	33
Actin, cytoplasmic 1 OS=Homo sapiens GN=ACTB PE=1 SV=1	sp|P60709|ACTB_HUMAN (+1)	42	10	31	15	23
Ceruloplasmin OS=Homo sapiens GN=CP PE=1 SV=1	sp|P00450|CERU_HUMAN	122	10	5	9	43
Immunoglobulin heavy variable 3-33 OS=Homo sapiens GN=IGHV3-33 PE=1 SV=2	sp|P01772|HV333_HUMAN	13	10	15	23	9
Platelet basic protein OS=Homo sapiens GN=PPBP PE=1 SV=3	sp|P02775|CXCL7_HUMAN	14	10	11	8	9
Ficolin-3 OS=Homo sapiens GN=FCN3 PE=1 SV=2	sp|O75636|FCN3_HUMAN	33	10	3	0	3
Filaggrin OS=Homo sapiens GN=FLG PE=1 SV=3	sp|P20930|FILA_HUMAN	435	9	22	2	15
Plakophilin-1 OS=Homo sapiens GN=PKP1 PE=1 SV=2	sp|Q13835|PKP1_HUMAN	83	9	9	2	15
Caspase-14 OS=Homo sapiens GN=CASP14 PE=1 SV=2	sp|P31944|CASPE_HUMAN	28	9	13	3	10
Immunoglobulin J chain OS=Homo sapiens GN=JCHAIN PE=1 SV=4	sp|P01591|IGJ_HUMAN	18	9	6	18	6
Immunoglobulin kappa variable 4-1 OS=Homo sapiens GN=IGKV4-1 PE=1 SV=1	sp|P06312|KV401_HUMAN	13	9	4	5	2

Arachidonate 12-lipoxygenase, 12R-type OS=Homo sapiens GN=ALOX12B PE=1 SV=1	sp|O75342|LX12B_HUMAN	80	9	6	0	7
Serum amyloid P-component OS=Homo sapiens GN=APCS PE=1 SV=2	sp|P02743|SAMP_HUMAN	25	8	34	67	55
Vitamin K-dependent protein S OS=Homo sapiens GN=PROS1 PE=1 SV=1	sp|P07225|PROS_HUMAN	75	8	40	36	63
Apolipoprotein(a) OS=Homo sapiens GN=LPA PE=1 SV=1	sp|P08519|APOA_HUMAN	501	8	15	45	6
Annexin A2 OS=Homo sapiens GN=ANXA2 PE=1 SV=2	sp|P07355|ANXA2_HUMAN	39	8	7	6	13
Small proline-rich protein 2E OS=Homo sapiens GN=SPRR2E PE=2 SV=2	sp|P22531|SPR2E_HUMAN	8	8	6	5	4
Apolipoprotein A-II OS=Homo sapiens GN=APOA2 PE=1 SV=1	sp|P02652|APOA2_HUMAN	11	8	2	8	0
Complement factor I OS=Homo sapiens GN=CFI PE=1 SV=2	sp|P05156|CFAI_HUMAN	66	7	43	96	117
Immunoglobulin kappa variable 3-20 OS=Homo sapiens GN=IGKV3-20 PE=1 SV=2	sp|P01619|KV320_HUMAN	13	7	9	17	6
Peroxiredoxin-1 OS=Homo sapiens GN=PRDX1 PE=1 SV=1	sp|Q06830|PRDX1_HUMAN	22	7	9	4	6
Alpha-1B-glycoprotein OS=Homo sapiens GN=A1BG PE=1 SV=4	sp|P04217|A1BG_HUMAN	54	7	4	7	9
Ig lambda-7 chain C region OS=Homo sapiens GN=IGLC7 PE=4 SV=2	sp|A0M8Q6|LAC7_HUMAN	11	7	0	11	7
Hepatocyte growth factor activator OS=Homo sapiens GN=HGFAC PE=1 SV=1	sp|Q04756|HGFA_HUMAN	71	6	43	13	27
Properdin OS=Homo sapiens GN=CFP PE=1 SV=2	sp|P27918|PROP_HUMAN	51	6	18	6	4
Protein Z-dependent protease inhibitor OS=Homo sapiens GN=SERPINA10 PE=1 SV=1	sp|Q9UK55|ZPI_HUMAN	51	6	14	11	2
Fructose-bisphosphate aldolase A OS=Homo sapiens GN=ALDOA PE=1 SV=2	sp|P04075|ALDOA_HUMAN	39	6	0	0	0
Immunoglobulin kappa variable 3-15 OS=Homo sapiens GN=IGKV3-15 PE=1 SV=2	sp|P01624|KV315_HUMAN	12	5	2	19	6
Immunoglobulin lambda variable 3-19 OS=Homo sapiens GN=IGLV3-19 PE=1 SV=2	sp|P01714|LV319_HUMAN	12	5	3	7	7
Immunoglobulin heavy variable 4-39 OS=Homo sapiens GN=IGHV4-39 PE=1 SV=2	sp|P01824|HV439_HUMAN (+2)	14	5	0	8	2
Neuroblast differentiation-associated protein AHNAK OS=Homo sapiens GN=AHNAK PE=1 SV=2	sp|Q09666|AHNK_HUMAN	629	5	3	0	3
Serum amyloid A-4 protein OS=Homo sapiens GN=SAA4 PE=1 SV=2	sp|P35542|SAA4_HUMAN	15	5	2	5	0
Protein S100-A14 OS=Homo sapiens GN=S100A14 PE=1 SV=1	sp|Q9HCY8|S10AE_HUMAN	12	5	2	0	2
Serum paraoxonase/arylesterase 1 OS=Homo sapiens GN=PON1 PE=1 SV=3	sp|P27169|PON1_HUMAN	40	4	6	15	15
Glycoprotein hormones alpha chain OS=Homo sapiens GN=CGA PE=1 SV=1	sp|P01215|GLHA_HUMAN	13	4	12	7	11
Gasdermin-A OS=Homo sapiens GN=GSDMA PE=1 SV=4	sp|Q96QA5|GSDMA_HUMAN	49	4	5	0	8
Gamma-glutamyl hydrolase OS=Homo sapiens GN=GGH PE=1 SV=2	sp|Q92820|GGH_HUMAN	36	4	7	6	5
Fatty acid-binding protein, epidermal OS=Homo sapiens GN=FABP5 PE=1 SV=3	sp|Q01469|FABP5_HUMAN	15	4	14	0	6
Gamma-glutamylcyclotransferase OS=Homo sapiens GN=GGCT PE=1 SV=1	sp|O75223|GGCT_HUMAN	21	4	11	0	3
Serpin B12 OS=Homo sapiens GN=SERPINB12 PE=1 SV=1	sp|Q96P63|SPB12_HUMAN	46	4	8	0	5
Galectin-7 OS=Homo sapiens GN=LGALS7 PE=1 SV=2	sp|P47929|LEG7_HUMAN	15	4	5	0	2
Peptidyl-prolyl cis-trans isomerase B OS=Homo sapiens GN=PPIB PE=1 SV=2	sp|P23284|PPIB_HUMAN	24	4	0	0	0
Protein-glutamine gamma-glutamyltransferase E OS=Homo sapiens GN=TGM3 PE=1 SV=4	sp|Q08188|TGM3_HUMAN	77	3	18	2	8
Immunoglobulin kappa variable 2-30 OS=Homo sapiens GN=IGKV2-30 PE=3 SV=2	sp|P06310|KV230_HUMAN	13	3	0	6	2
Desmocollin-1 OS=Homo sapiens GN=DSC1 PE=1 SV=2	sp|Q08554|DSC1_HUMAN	100	3	2	0	12

Mannose-binding protein C OS=Homo sapiens GN=MBL2 PE=1 SV=2	sp|P11226|MBL2_HUMAN	26	3	3	8	4
Immunoglobulin lambda variable 2-23 OS=Homo sapiens GN=IGLV2-23 PE=1 SV=2	sp|P01705|LV223_HUMAN	12	3	3	3	0
Immunoglobulin lambda variable 1-47 OS=Homo sapiens GN=IGLV1-47 PE=1 SV=2	sp|P01700|LV147_HUMAN	12	3	0	4	2
Angiotensinogen OS=Homo sapiens GN=AGT PE=1 SV=1	sp|P01019|ANGT_HUMAN	53	3	5	0	2
Heat shock protein HSP 90-beta OS=Homo sapiens GN=HSP90AB1 PE=1 SV=4	sp|P08238|HS90B_HUMAN	83	3	3	0	0
Histone H2B type 1-K OS=Homo sapiens GN=HIST1H2BK PE=1 SV=3	sp|O60814|H2B1K_HUMAN (+7)	14	3	0	3	2
Thrombospondin-1 OS=Homo sapiens GN=THBS1 PE=1 SV=2	sp|P07996|TSP1_HUMAN	129	3	2	0	3
Protein S100-A16 OS=Homo sapiens GN=S100A16 PE=1 SV=1	sp|Q96FQ6|S10AG_HUMAN	12	3	0	0	0
Elongation factor 1-alpha 1 OS=Homo sapiens GN=EEF1A1 PE=1 SV=1	sp|P68104|EF1A1_HUMAN (+1)	50	3	0	0	2
ADP-ribosylation factor 3 OS=Homo sapiens GN=ARF3 PE=1 SV=2	sp|P61204|ARF3_HUMAN (+2)	21	3	0	0	0
Choriogonadotropin subunit beta 3 OS=Homo sapiens GN=CGB3 PE=1 SV=1	sp|P0DN86|CGB3_HUMAN (+2)	18	2	17	9	15
Serpin B3 OS=Homo sapiens GN=SERPINB3 PE=1 SV=2	sp|P29508|SPB3_HUMAN	45	2	22	0	0
Immunoglobulin kappa variable 1-17 OS=Homo sapiens GN=IGKV1-17 PE=1 SV=2	sp|P01599|KV117_HUMAN	13	2	0	3	3
Protein S100-A7 OS=Homo sapiens GN=S100A7 PE=1 SV=4	sp|P31151|S10A7_HUMAN	11	2	8	2	3
Neutrophil defensin 3 OS=Homo sapiens GN=DEFA3 PE=1 SV=1	sp|P59666|DEF3_HUMAN	10 k	2	6	2	2
Cathepsin D OS=Homo sapiens GN=CTSD PE=1 SV=1	sp|P07339|CATD_HUMAN	45	2	9	0	5
Insulin-like growth factor-binding protein 6 OS=Homo sapiens GN=IGFBP6 PE=1 SV=1	sp|P24592|IBP6_HUMAN	25	2	6	0	2
Prolactin-inducible protein OS=Homo sapiens GN=PIP PE=1 SV=1	sp|P12273|PIP_HUMAN	17	2	2	0	0
Ig delta chain C region OS=Homo sapiens GN=IGHD PE=1 SV=2	sp|P01880|IGHD_HUMAN	42	2	0	7	6
Glutathione peroxidase 3 OS=Homo sapiens GN=GPX3 PE=1 SV=2	sp|P22352|GPX3_HUMAN	26	2	0	2	2
Zinc-alpha-2-glycoprotein OS=Homo sapiens GN=AZGP1 PE=1 SV=2	sp|P25311|ZA2G_HUMAN	34	2	6	0	4
14-3-3 protein zeta/delta OS=Homo sapiens GN=YWHAZ PE=1 SV=1	sp|P63104|1433Z_HUMAN	28	2	2	5	2
Cystatin-A OS=Homo sapiens GN=CSTA PE=1 SV=1	sp|P01040|CYTA_HUMAN	11	2	4	0	0
Lipocalin-1 OS=Homo sapiens GN=LCN1 PE=1 SV=1	sp|P31025|LCN1_HUMAN	19	2	4	2	2
Catalase OS=Homo sapiens GN=CAT PE=1 SV=3	sp|P04040|CATA_HUMAN	60	2	3	0	4
Metalloproteinase inhibitor 2 OS=Homo sapiens GN=TIMP2 PE=1 SV=2	sp|P16035|TIMP2_HUMAN	24	2	3	3	0
Extracellular superoxide dismutase [Cu-Zn] OS=Homo sapiens GN=SOD3 PE=1 SV=2	sp|P08294|SODE_HUMAN	26	2	3	0	3
Tubulin alpha-1B chain OS=Homo sapiens GN=TUBA1B PE=1 SV=1	sp|P68363|TBA1B_HUMAN (+2)	50	2	0	0	2
Centromere-associated protein E OS=Homo sapiens GN=CENPE PE=1 SV=2	sp|Q02224|CENPE_HUMAN	316	2	0	0	0
Immunoglobulin lambda variable 7-43 OS=Homo sapiens GN=IGLV7-43 PE=3 SV=2	sp|P04211|LV743_HUMAN	12	2	2	2	0
Histone H4 OS=Homo sapiens GN=HIST1H4A PE=1 SV=2	sp|P62805|H4_HUMAN	11	2	0	0	2
Heat shock cognate 71 kDa protein OS=Homo sapiens GN=HSPA8 PE=1 SV=1	sp|P11142|HSP7C_HUMAN	71	2	2	0	2
Protein POF1B OS=Homo sapiens GN=POF1B PE=1 SV=3	sp|Q8WVV4|POF1B_HUMAN	68	2	2	0	3
L-lactate dehydrogenase A chain OS=Homo sapiens GN=LDHA PE=1 SV=2	sp|P00338|LDHA_HUMAN	37	2	0	0	0

Pyruvate kinase PKM OS=Homo sapiens GN=PKM PE=1 SV=4	sp|P14618|KPYM_HUMAN	58	2	0	0	0
Fibrinogen beta chain OS=Homo sapiens GN=FGB PE=1 SV=2	sp|P02675|FIBB_HUMAN	56	0	36	263	245
Fibrinogen gamma chain OS=Homo sapiens GN=FGG PE=1 SV=3	sp|P02679|FIBG_HUMAN	52	0	44	233	215
Complement component C7 OS=Homo sapiens GN=C7 PE=1 SV=2	sp|P10643|CO7_HUMAN	94	0	0	0	120
Keratin, type II cytoskeletal 4 OS=Homo sapiens GN=KRT4 PE=1 SV=4	sp|P19013|K2C4_HUMAN	57	0	10	43	39
Keratin, type I cytoskeletal 13 OS=Homo sapiens GN=KRT13 PE=1 SV=4	sp|P13646|K1C13_HUMAN	50	0	20	34	70
Complement C1r subcomponent OS=Homo sapiens GN=C1R PE=1 SV=2	sp|P00736|C1R_HUMAN	80	0	9	10	48
Complement component C6 OS=Homo sapiens GN=C6 PE=1 SV=3	sp|P13671|CO6_HUMAN	105	0	0	0	75
Selenoprotein P OS=Homo sapiens GN=SEPP1 PE=1 SV=3	sp|P49908|SEPP1_HUMAN	43	0	24	6	5
Immunoglobulin heavy variable 3-15 OS=Homo sapiens GN=IGHV3-15 PE=3 SV=1	sp|A0A0B4J1V0|HV315_HUMAN	13	0	0	7	0
Keratin, type II cytoskeletal 2 oral OS=Homo sapiens GN=KRT76 PE=1 SV=2	sp|Q01546|K22O_HUMAN	66	0	17	0	0
Ig gamma-4 chain C region OS=Homo sapiens GN=IGHG4 PE=1 SV=1	sp|P01861|IGHG4_HUMAN	36	0	0	31	0
Lactotransferrin OS=Homo sapiens GN=LTF PE=1 SV=6	sp|P02788|TRFL_HUMAN	78	0	20	0	5
Galectin-3-binding protein OS=Homo sapiens GN=LGALS3BP PE=1 SV=1	sp|Q08380|LG3BP_HUMAN	65	0	15	0	5
Transthyretin OS=Homo sapiens GN=TTR PE=1 SV=1	sp|P02766|TTHY_HUMAN	16	0	2	6	4
Coagulation factor V OS=Homo sapiens GN=F5 PE=1 SV=4	sp|P12259|FA5_HUMAN	252	0	5	0	10
Annexin A1 OS=Homo sapiens GN=ANXA1 PE=1 SV=2	sp|P04083|ANXA1_HUMAN	39	0	2	11	9
Phosphatidylinositol-glycan-specific phospholipase D OS=Homo sapiens GN=GPLD1 PE=1 SV=3	sp|P80108|PHLD_HUMAN	92	0	2	0	18
Polyubiquitin-B OS=Homo sapiens GN=UBB PE=1 SV=1	sp|P0CG47|UBB_HUMAN (+3)	26	0	6	0	0
Alpha-amylase 1 OS=Homo sapiens GN=AMY1A PE=1 SV=2	sp|P04745|AMY1_HUMAN	58	0	0	13	0
F-box only protein 50 OS=Homo sapiens GN=NCCRP1 PE=1 SV=1	sp|Q6ZVX7|FBX50_HUMAN	31	0	2	0	4
Apolipoprotein L1 OS=Homo sapiens GN=APOL1 PE=1 SV=5	sp|O14791|APOL1_HUMAN	44	0	0	12	0
C4b-binding protein beta chain OS=Homo sapiens GN=C4BPB PE=1 SV=1	sp|P20851|C4BPB_HUMAN	28	0	3	3	7
Complement factor H-related protein 4 OS=Homo sapiens GN=CFHR4 PE=1 SV=3	sp|Q92496|FHR4_HUMAN	65	0	8	0	2
Neuropilin-1 OS=Homo sapiens GN=NRP1 PE=1 SV=3	sp|O14786|NRP1_HUMAN	103	0	8	0	2
L-lactate dehydrogenase B chain OS=Homo sapiens GN=LDHB PE=1 SV=2	sp|P07195|LDHB_HUMAN	37	0	2	0	6
Alpha-2-antiplasmin OS=Homo sapiens GN=SERPINF2 PE=1 SV=3	sp|P08697|A2AP_HUMAN	55	0	3	0	4
Ribonuclease pancreatic OS=Homo sapiens GN=RNASE1 PE=1 SV=4	sp|P07998|RNAS1_HUMAN	18	0	11	0	0
Pregnancy-specific beta-1-glycoprotein 6 OS=Homo sapiens GN=PSG6 PE=2 SV=1	sp|Q00889|PSG6_HUMAN	49	0	6	0	6
Neutrophil gelatinase-associated lipocalin OS=Homo sapiens GN=LCN2 PE=1 SV=2	sp|P80188|NGAL_HUMAN	23	0	5	0	5
Coagulation factor XIII B chain OS=Homo sapiens GN=F13B PE=1 SV=3	sp|P05160|F13B_HUMAN	76	0	0	0	11
ATP synthase subunit alpha, mitochondrial OS=Homo sapiens GN=ATP5A1 PE=1 SV=1	sp|P25705|ATPA_HUMAN	60	0	0	3	3
Arginase-1 OS=Homo sapiens GN=ARG1 PE=1 SV=2	sp|P05089|ARGI1_HUMAN	35	0	2	0	2
Vitamin D-binding protein OS=Homo sapiens GN=GC PE=1 SV=1	sp|P02774|VTDB_HUMAN	53	0	7	0	2

Retinol-binding protein 4 OS=Homo sapiens GN=RBP4 PE=1 SV=3	sp|P02753|RET4_HUMAN	23	0	0	4	7
Hemoglobin subunit beta OS=Homo sapiens GN=HBB PE=1 SV=2	sp|P68871|HBB_HUMAN	16	0	0	0	2
Immunoglobulin kappa variable 2-28 OS=Homo sapiens GN=IGKV2-28 PE=3 SV=1	sp|A0A075B6P5|KV228_HUMAN (+2)	13	0	0	0	3
Calmodulin-like protein 5 OS=Homo sapiens GN=CALML5 PE=1 SV=2	sp|Q9NZT1|CALL5_HUMAN	16	0	3	0	0
Small proline-rich protein 3 OS=Homo sapiens GN=SPRR3 PE=1 SV=2	sp|Q9UBC9|SPRR3_HUMAN	18	0	0	6	4
Immunoglobulin lambda variable 2-8 OS=Homo sapiens GN=IGLV2-8 PE=1 SV=2	sp|P01709|LV208_HUMAN	12	0	0	5	0
Alpha-2-HS-glycoprotein OS=Homo sapiens GN=AHSG PE=1 SV=1	sp|P02765|FETUA_HUMAN	39	0	0	0	3
Immunoglobulin heavy variable 1-46 OS=Homo sapiens GN=IGHV1-46 PE=1 SV=2	sp|P01743|HV146_HUMAN	13	0	0	4	0
Suprabasin OS=Homo sapiens GN=SBSN PE=1 SV=2	sp|Q6UWP8|SBSN_HUMAN	61	0	2	0	2
P-selectin OS=Homo sapiens GN=SELP PE=1 SV=3	sp|P16109|LYAM3_HUMAN	91	0	4	0	2
Cartilage acidic protein 1 OS=Homo sapiens GN=CRTAC1 PE=1 SV=2	sp|Q9NQ79|CRAC1_HUMAN	71	0	0	0	3
Cytoplasmic dynein 2 heavy chain 1 OS=Homo sapiens GN=DYNC2H1 PE=1 SV=4	sp|Q8NCM8|DYHC2_HUMAN	493	0	2	0	0
Protein S100-A11 OS=Homo sapiens GN=S100A11 PE=1 SV=2	sp|P31949|S10AB_HUMAN	12	0	0	2	0
Heat shock 70 kDa protein 1A OS=Homo sapiens GN=HSPA1A PE=1 SV=1	sp|P0DMV8|HS71A_HUMAN (+2)	70	0	3	0	0
Hydroperoxide isomerase ALOXE3 OS=Homo sapiens GN=ALOXE3 PE=1 SV=1	sp|Q9BYJ1|LOXE3_HUMAN	81	0	0	0	2
Transmembrane glycoprotein NMB OS=Homo sapiens GN=GPNMB PE=1 SV=2	sp|Q14956|GPNMB_HUMAN	64	0	2	0	0
Polymeric immunoglobulin receptor OS=Homo sapiens GN=PIGR PE=1 SV=4	sp|P01833|PIGR_HUMAN	83	0	2	0	0
Tropomyosin alpha-4 chain OS=Homo sapiens GN=TPM4 PE=1 SV=3	sp|P67936|TPM4_HUMAN	29	0	2	0	0
A disintegrin and metalloproteinase with thrombospondin motifs 13 OS=Homo sapiens GN=ADAMTS13 PE=1 SV=1	sp|Q76LX8|ATS13_HUMAN	154	0	2	0	0
Tenascin-X OS=Homo sapiens GN=TNXB PE=1 SV=4	sp|P22105|TENX_HUMAN	458	0	0	0	4
Immunoglobulin lambda variable 1-51 OS=Homo sapiens GN=IGLV1-51 PE=1 SV=2	sp|P01701|LV151_HUMAN	12	0	0	3	0
Thioredoxin OS=Homo sapiens GN=TXN PE=1 SV=3	sp|P10599|THIO_HUMAN	12	0	2	0	0
Hemoglobin subunit alpha OS=Homo sapiens GN=HBA1 PE=1 SV=2	sp|P69905|HBA_HUMAN	15	0	0	0	2
Insulin-like growth factor-binding protein 2 OS=Homo sapiens GN=IGFBP2 PE=1 SV=2	sp|P18065|IBP2_HUMAN	35	0	2	0	0
Insulin-like growth factor-binding protein 4 OS=Homo sapiens GN=IGFBP4 PE=1 SV=2	sp|P22692|IBP4_HUMAN	28	0	3	0	0
Pregnancy-specific beta-1-glycoprotein 4 OS=Homo sapiens GN=PSG4 PE=2 SV=3	sp|Q00888|PSG4_HUMAN	47	0	7	0	0
Keratin, type I cytoskeletal 18 OS=Homo sapiens GN=KRT18 PE=1 SV=2	sp|P05783|K1C18_HUMAN	48 k	0	3	0	0
Peptidoglycan recognition protein 1 OS=Homo sapiens GN=PGLYRP1 PE=1 SV=1	sp|O75594|PGRP1_HUMAN	22	0	2	0	0
Attractin OS=Homo sapiens GN=ATRN PE=1 SV=2	sp|O75882|ATRN_HUMAN	159	0	0	0	2
Inter-alpha-trypsin inhibitor heavy chain H3 OS=Homo sapiens GN=ITIH3 PE=1 SV=2	sp|Q06033|ITIH3_HUMAN	100	0	0	0	2
Lysosome-associated membrane glycoprotein 1 OS=Homo sapiens GN=LAMP1 PE=1 SV=3	sp|P11279|LAMP1_HUMAN	45	0	2	0	0
Mucin-5B OS=Homo sapiens GN=MUC5B PE=1 SV=3	sp|Q9HC84|MUC5B_HUMAN	596	0	0	2	0
Collagen alpha-1(III) chain OS=Homo sapiens GN=COL3A1 PE=1 SV=4	sp|P02461|CO3A1_HUMAN	139	0	0	0	3

Alpha-2-macroglobulin-like protein 1 OS=Homo sapiens GN=A2ML1 PE=1 SV=3	sp|A8K2U0|A2ML1_HUMAN	161	0	2	0	0
Protein sel-1 homolog 3 OS=Homo sapiens GN=SEL1L3 PE=1 SV=2	sp|Q68CR1|SE1L3_HUMAN	129	0	0	0	2
Pregnancy-specific beta-1-glycoprotein 1 OS=Homo sapiens GN=PSG1 PE=1 SV=1	sp|P11464|PSG1_HUMAN	47	0	0	0	8
